# Diabetes in Cystic Fibrosis: Multicenter Screening Results Based on Current Guidelines

**DOI:** 10.1371/journal.pone.0081545

**Published:** 2013-12-06

**Authors:** Nicole Scheuing, Reinhard W. Holl, Gerd Dockter, Katharina Fink, Sibylle Junge, Lutz Naehrlich, Christina Smaczny, Doris Staab, Gabriela Thalhammer, Silke van Koningsbruggen-Rietschel, Manfred Ballmann

**Affiliations:** 1 Institute of Epidemiology and Medical Biometry, Central Institute for Biomedical Engineering, University of Ulm, Ulm, Germany; 2 Cystic Fibrosis Centre, Saarland University Hospital for Pediatric and Adolescent Medicine, Homburg/Saar, Germany; 3 Clinic for Pediatric Pneumology and Neonatology, Hannover Medical School, Hannover, Germany; 4 Department of Pediatrics, Justus-Liebig University Giessen, Giessen, Germany; 5 Medical Clinic I, Pneumology and Allergology, Johann Wolfgang Goethe University, Frankfurt/Main, Germany; 6 Division of Pulmonology and Immunology, Department of Pediatrics, Charité Berlin, Berlin, Germany; 7 Department for Pediatric Pulmonology and Allergology, Medical University of Graz, Graz, Austria; 8 Cystic Fibrosis Centre Cologne, Childreńs Hospital, University of Cologne, Cologne, Germany; 9 Department of Pediatric Pulmonology, St. Josef Hospital Pediatric Clinic, Ruhr University Bochum, Bochum, Germany; University of Tübingen, Germany

## Abstract

**Background:**

Published estimates on age-dependent frequency of diabetes in cystic fibrosis (CF) vary widely, and are based mostly on older data. However, CF treatment and prevention of comorbidities changed over recent years. In many studies, definition of cystic fibrosis-related diabetes (CFRD) is not in line with current guideline recommendations. Therefore, we evaluated age-dependent occurrence of glucose abnormalities and associated risk factors in CF patients who participated in a multicenter screening program using oral glucose tolerance tests (OGTT).

**Methods:**

Between 2001 and 2010, 43 specialized CF centers from Germany and Austria serially performed 5,179 standardized OGTTs in 1,658 clinically stable, non-pregnant CF patients with no prior steroid medication or lung transplantation. Age-dependent occurrence of impaired fasting glucose (IFG), impaired glucose tolerance (IGT), IFG+IGT, one (DGT) or two consecutive (CFRD) diabetic OGTTs was analyzed, using Kaplan Meier curves. Cox proportional-hazards models were created to elucidate the influence of sex or underweight.

**Results:**

At baseline/last OGTT, median age was 15.9 years/18.2 years and 30.6%/31.8% of patients were underweight. 25% of patients showed IFG at age 14.3 years; IGT at age 16.3 years; IFG+IGT combined at age 17.7 years. DGT was observed in 25% of patients at age 22.6 years; CFRD at age 34.5 years. Females had a 3.54 [95% CI 1.23–10.18] times higher risk for CFRD; risk for DGT was 2.21 [1.22–3.98] times higher. Underweight was a risk factor for IGT (HR [95% CI]: 1.38 [1.11–1.71]) and IFG+IGT (1.43 [1.11–1.83]), and in males also for DGT (1.49 [1.09–2.04]).

**Conclusions/Significance:**

If confirmation of diabetes by a second test is required, as recommended in current guidelines, age at CFRD diagnosis was higher compared to most previous studies. However, known risk factors for glucose abnormalities in CF were confirmed. Confirmation of diabetic OGT by a repeat test is important for a consistent diagnosis of CFRD.

## Introduction

Cystic fibrosis (CF) is the most frequent autosomal recessive disease in Caucasians. Due to improvements in medical and nutritional therapy, life expectancy in CF increased over the last decades. In parallel, CF-related comorbidities became more frequent. The most common comorbidity is cystic fibrosis-related diabetes (CFRD). It occurs in about 2% of children, 19% of adolescents and 40–50% of adults [1]. CFRD shares features with insulin-deficient type 1 and with insulin-resistant type 2 diabetes [2], [3], but it is a separate clinical entity. The primary cause of CFRD is insulin insufficiency due to reduced beta cell mass [4]. However, contrary to type 1 diabetes mellitus, insulin secretion is never totally absent in CFRD because destruction of beta cells is incomplete. As in type 2 diabetes mellitus, insulin resistance also plays a role in CFRD, although it is usually mild and its degree varies with infection status and steroid therapy. Genetic predisposition for CFRD by the underlying CF gene defect as well as by moderator genes is discussed [4].

Early CFRD diagnosis is important because this complication is associated with a series of negative effects on the course of CF. In CFRD patients, e.g. frequency of infections, decline of pulmonary function, weight loss and growth impairment, occurrence of microvascular complications as well as mortality are higher compared to non-diabetic CF patients [4]–[6]. However, CFRD development is mostly asymptomatic [7] and thereby early diagnosis is difficult. Thus, the current international guidelines recommend annual screening for CFRD in all CF patients aged ten years or older [2]. CFRD in patients younger than ten years is rare [8]. According to current guidelines, the screening tool of choice should be the oral glucose tolerance test (OGTT) [2]. Other screening parameters like hemoglobin A1c (HbA1c), fructosamine, urine glucose or random glucose levels should not be used because of low sensitivity in the CF population [2]. Continuous glucose monitoring is also currently not recommended for screening [2]. Even though variability in OGTT exists, several previous studies support the use of OGTT to identify patients with CFRD [7], [9], [10]. In a small recent study with OGTTs, no relevant difference in glucose tolerance during CF exacerbations compared to clinical stability was found in most patients [11].

Based on these findings and the guideline recommendation, we used OGTT in one of the largest screening programs for CFRD. Previous screening studies are based mostly on older data from the eighties and nineties. However, treatment of CF and prevention of comorbidities changed over recent years. Therefore, the primary aim of the present analysis was to investigate the occurrence of CFRD, or abnormalities in glucose metabolism, depending on age, gender and nutritional status among CF patients screened between the years 2001 and 2010. In contrast to most previous studies, we used a clear definition for CFRD in compliance with current international guidelines. The results were compared to earlier findings. A second goal was to analyze, whether fasting blood glucose (FBG) levels or blood glucose levels 2 h post-glucose challenge (2 h-BG) rise earlier in CF. This has impact on the choice of treatment.

## Materials and Methods

### 2.1 Ethics Statement

The study has been approved by the ethical committees of the University of Ulm and the Hannover Medical School. Written informed consent was obtained from each patient, or his guardian if aged <18 years.

### 2.2 Study Subjects

Patients were recruited during a longitudinal, prospective, multicenter study on ‘Early Diagnosis of Diabetes Mellitus in Patients with Cystic Fibrosis’ (Trial No. NCT00662714) carried out between 2001 and 2010. 43 specialized centers (40 German, 3 Austrian) performed standardized OGTTs to screen serially for CFRD starting at age 10. In case of a pathological OGTT, testing was repeated: according to study protocol, a diabetic OGTT was to be repeated after 4 to 6 weeks and an OGTT revealing impaired glucose tolerance (IGT) or impaired fasting glucose (IFG) was to be repeated after 6 months. For each CF patient screened, demographic data, OGTT results (at least FBG and 2 h-BG) and further clinical characteristics were documented anonymously in standardized form in a central database.

**Figure 1:**
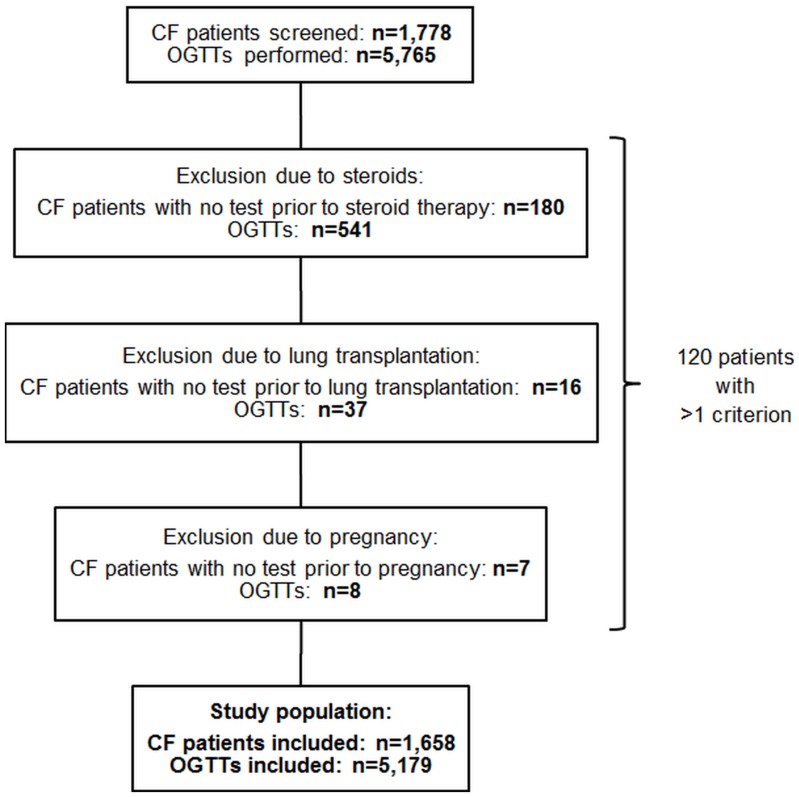
Selection of study population. Oral glucose tolerance tests of patients with prior steroid therapy or lung transplantation were excluded as well as tests in pregnant patients. The final study population comprised 5,179 oral glucose tolerance tests performed in 1,658 cystic fibrosis patients. Abbreviations: CF = cystic fibrosis, OGTT = oral glucose tolerance test.

Until the end of the study, the database comprised plausible data on 5,765 OGTTs performed in 1,778 CF patients. All OGTTs of patients with prior steroid therapy or lung transplantation were excluded from analysis (Figure 1). If patients were pregnant at the time of screening, OGTTs were also excluded (Figure 1).

Finally, 5,179 OGTTs of 1,658 patients were included in the present analysis (Figure 1). 4,921 OGTTs were performed in 1,513 German CF patients and 258 OGTTs were from 145 Austrian patients. 1,129 patients had at least two OGTTs. In 54 (15.9%) out of 340 patients with a diabetic OGTT no further OGTT was performed within the study period. Besides the end of the study, additional reasons for lack of a second OGTT after a diabetic OGTT might be the occurrence of exclusion criteria after the first test or no further participation of patients in the study.

### 2.3 Procedure and Interpretation of OGTT

At the time of OGTT, CF patients were clinically stable, with no symptoms of acute infections and no exacerbations of chronic infections, and consumed a high energy diet with high carbohydrate intake. Patients with aggressive nutritional supplementation (e.g. enteral tube feeds) were excluded from screening. OGTTs were performed according to WHO guidelines [12], as described previously [13], [14]. In 2003, the ADA lowered the threshold for normal fasting plasma glucose from 6.1 mmol/l to 5.6 mmol/l [15]. Therefore, WHO criteria [12] modified by recommendations of ADA [15] were used for the interpretation of OGTT results. Normal glucose tolerance (NGT) was defined as fasting venous or capillary plasma glucose <5.6 mmol/l and plasma glucose 2 h post-glucose challenge <7.8 mmol/l for venous plasma or <8.9 mmol/l for capillary plasma. Fasting venous or capillary plasma glucose between ≥5.6–<7.0 mmol/l was defined as IFG. Plasma glucose levels 2 h post-glucose challenge between ≥7.8–<11.1 mmol/l for venous plasma or ≥8.9–<12.2 mmol/l for capillary plasma were defined as IGT. A diabetic glucose tolerance (DGT) was defined as venous or capillary plasma glucose ≥7.0 mmol/l or plasma glucose 2 h post-glucose challenge ≥11.1 mmol/l for venous plasma or ≥12.2 mmol/l for capillary plasma. For glucose levels measured in whole blood samples, the individual cut-offs were converted according to WHO [12].

CFRD was diagnosed, if two consecutive OGTTs showed a diabetic glucose tolerance [2].

### 2.4 Anthropometry

For patients ≤18 years, weight, height and body mass index standard deviation scores (BMI-SDS) were calculated using contemporary representative national reference data from the KiGGS study [16]. Underweight was defined according to international guidelines [17], [18]: for children and adolescents (≤18 years), BMI values below the 10th percentile were classified as underweight and for adults (>18 years), underweight was defined as BMI<19 kg/m^2^.

### 2.5 Statistical Analyses

The statistical package SAS 9.3 (Statistical Analysis Software, SAS Institute Inc., Cary, NC, USA) was used. Descriptive statistics were carried out for demographic data/diabetes symptoms at baseline and at last available OGTT. Results are presented as median with lower (Q1) and upper quartile (Q3), or as percentage (%).

Age-dependent occurrence of a pathological OGTT (at least IFG, IGT, IFG+IGT, DGT) and of a diagnosis of CFRD were analyzed using Kaplan Meier curves with censoring on patients who had no occurrence of the respective endpoint during their individual observation period, or required systemic steroid medication or lung transplantation. To evaluate gender differences, Kaplan Meier curves were stratified. As some gender-specific Kaplan Meier curves missed proportionality, comparisons were made using Wilcoxon test, which focuses especially on early events.

The question, whether FBG or 2 h-BG levels rise earlier in CF was investigated. Kaplan Meier curves were created for patient’s age at occurrence of FBG ≥5.6 mmol/l, ≥6.1 mmol/l, ≥7.0 mmol/l, ≥7.8 mmol/l or 2 h-BG ≥7.8 mmol/l, ≥11.1 mmol/l. The thresholds were taken from different definitions for abnormal glucose levels according to ADA 2003, WHO 1999 and WHO 1980 [12], [15], [19].

Hazard ratios (HR) with 95% confidence intervals (CI) for the influence of sex or underweight on the development of a pathological OGTT or CFRD were calculated using multivariable Cox proportional-hazards models with age as underlying time metric. The risk factors ‘sex’ and ‘underweight’ were included as dichotomous variables in each model. Proportional hazards assumption and functional form of covariates were assessed by inspection of martingale residuals and by supremum test. Based on the results of martingale residuals, supremum test, Akaike’s Information Criteria (AIC) and gender-specific Kaplan Meier curves, interaction terms for ‘underweight with sex’ as well as ‘age with sex’ were added to each model. Adjustments for ties were made by Breslow method.

Two-sided p-values <0.05 were considered statistically significant.

## Results

### 3.1 Study Subjects

Table 1 shows demographics and diabetes symptoms of subjects included in the present analysis. Between baseline and last available OGTT, no clinically relevant differences could be observed (Table 1). In patients with at least two OGTTs, median time interval between first and last OGTT was 3.1 [Q1; Q3: 1.5; 5.0] years. A diabetic OGTT was confirmed by a second diabetic OGTT on average within 8.1 [5.1; 27.9] weeks.

**Table 1:** Demographics and symptoms of the study population at baseline and at last available OGTT.

Clinical values		Baseline OGTT	Last available OGTT
Number of patients		1,658	1,658
Male/Female, %		52.4/47.6	52.4/47.6
Age, years		15.9 [11.7; 22.6]	18.2 [14.6; 25.1]
Height, cm/SDS			
	>18 years	170.0 [163.0; 177.0] (n = 650)	170.0 [163.0; 177.0] (n = 854)
	≤18 years	−0.82 [−1.57; −0.01] (n = 996)	−0.81 [−1.59; −0.01] (n = 770)
Weight, kg/SDS			
	>18 years	58.0 [51.4; 65.5] (n = 650)	58.5 [51.6; 66.0] (n = 854)
	≤18 years	−1.05 [−1.77; −0.32] (n = 995)	−1.09 [−1.82; −0.32] (n = 772)
Body mass index, kg·m^−^^2^/SDS			
	>18 years	20.2 [18.6; 22.1] (n = 650)	20.2 [18.6; 22.2] (n = 854)
	≤18 years	−0.80 [−1.44; −0.19] (n = 995)	−0.83 [−1.51; −0.16] (n = 769)
Underweight, %			
	All	30.6 (n = 1,645)	31.8 (n = 1,623)
	>18 years	31.1 (n = 650)	30.6 (n = 854)
	≤18 years	30.3 (n = 995)	33.2 (n = 769)
Diabetes symptoms, %			
	Weight loss	1.6	2.1
	Polyuria	0.5	0.7
	Polydipsia	0.3	0.3
	Nycturia	0.2	0.5
	Fatigue/loss of power	2.2	3.3

Data are median with lower and upper quartile for continuous variables, and percentage for dichotomous variables. For patients ≤18 years, standard deviation scores are shown for height, weight and body mass index. Weight and height measurements (and thereby body mass index, percentage underweight) were missing in few patients. Abbreviations: OGTT = oral glucose tolerance test, SDS = standard deviation score.

Underweight was present in about one third of CF patients at baseline/last OGTT (Table 1). In adult CF patients, no clinically relevant difference between genders existed. At baseline (last) OGTT, 30.7% (30.6%) of females and 31.5% (30.5%) of males had a BMI<19 kg/m^2^. In children and adolescents, boys showed a poorer nutritional state than girls. 31.8% (34.2%) of boys and 28.5% (32.1%) of girls had a BMI below the 10th percentile at baseline (last) OGTT.

Descriptive statistics for German or Austrian patients separately showed no relevant differences for demographic data and frequencies of diabetes symptoms (data not shown).

Patients, whose OGTTs were excluded from the analysis due to prior steroid medication or lung transplantation were older (20.6 [14.1; 30.0] years and 29.3 [23.4; 35.9] years), with a higher percentage of males (57.2% and 56.3%) compared to the study population. Underweight was present in 46.1% of patients with prior steroids and 31.3% of patients with prior lung transplantation. Patients excluded due to pregnancy were also older (23.7 [22.2; 33.1] years) than the study population and 14.3% of pregnant patients were underweight.

### 3.2 Abnormalities in Glucose Metabolism

**Figure 2:**
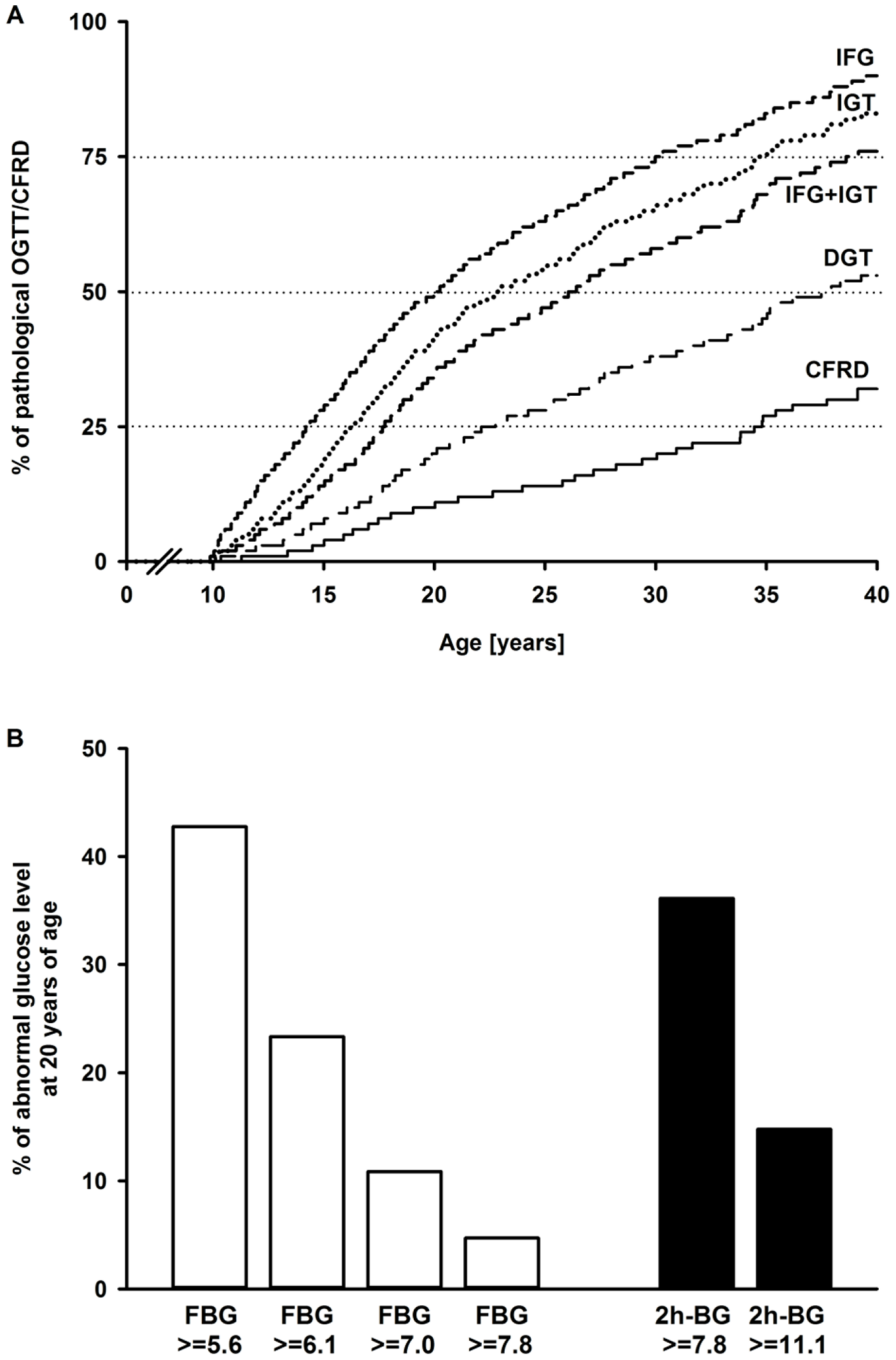
Kaplan Meier curves for abnormal glucose tolerance and percentage abnormal glucose levels at 20 years. (A) Age at first occurrence of a pathological oral glucose tolerance test or a diagnosis of cystic fibrosis-related diabetes. With increasing age, abnormalities in glucose tolerance became more frequent. Abbreviations: CFRD = cystic fibrosis-related diabetes, DGT = diabetic glucose tolerance, IFG = impaired fasting glucose, IGT = impaired glucose tolerance, OGTT = oral glucose tolerance test. (B) Depending on thresholds (in mmol/l) used, fasting blood glucose or blood glucose levels 2 h post-glucose challenge rise earlier in cystic fibrosis. Abbreviations: FBG = fasting blood glucose, 2 h-BG = blood glucose levels 2 h post-glucose challenge.

With increasing age, abnormalities of glucose tolerance in CF became more frequent (Figure 2A). Less severe glucose intolerance occurred earlier in life and showed higher frequency (Figure 2A).

In 20-year-old patients with abnormal glucose levels, the frequency of elevated FBG (≥5.6 mmol/l) was higher compared to the frequency of elevated 2 h-BG (≥7.8 mmol/l) (Figure 2B). If 6.1 mmol/l was used as cut-off for elevated FBG, the frequency was lower compared to 2 h-BG ≥7.8 mmol/l (Figure 2B). Because each category of glucose abnormality increased proportionally with age (Figure 2A), these relationships are not limited to 20-year-old patients.

**Figure 3:**
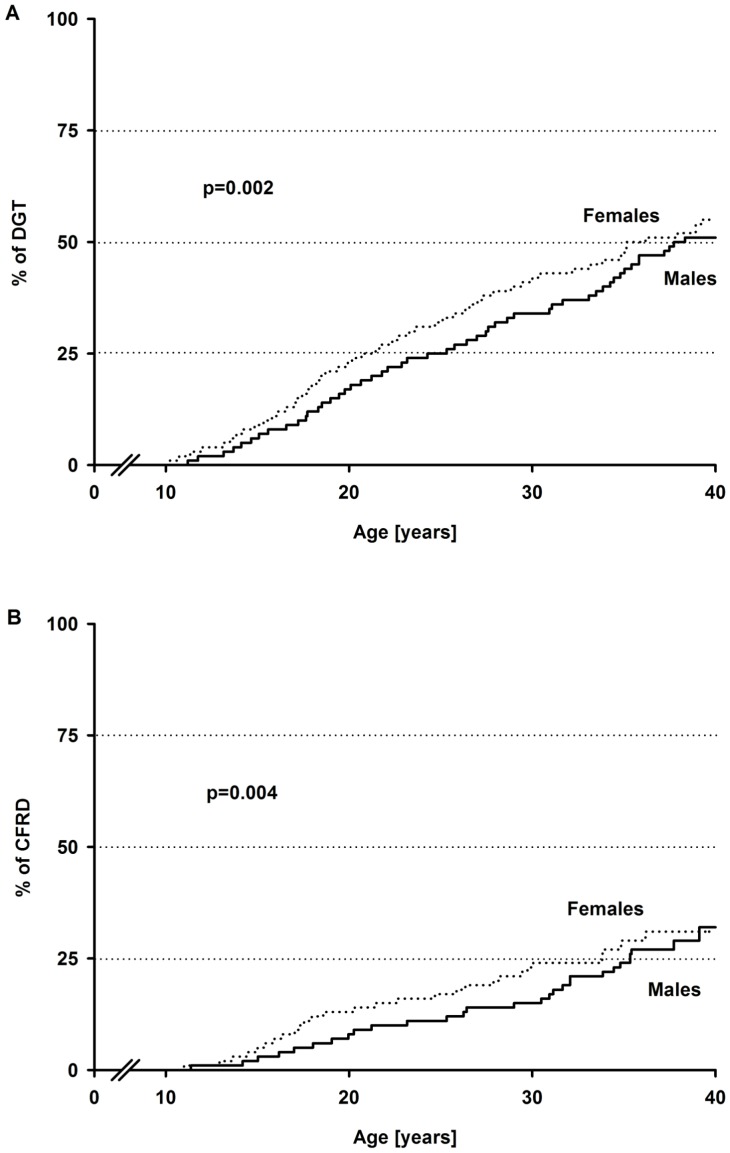
Gender-specific Kaplan Meier curves for a diabetic glucose tolerance and cystic fibrosis-related diabetes. A diabetic glucose tolerance (A) and cystic fibrosis-related diabetes (B) occurred earlier in females compared to males. Comparisons were made using Wilcoxon test. Abbreviations: DGT = diabetic glucose tolerance, CFRD = cystic fibrosis-related diabetes.

Gender-specific Kaplan Meier curves revealed a significantly earlier occurrence of DGT (Figure 3A) or CFRD (Figure 3B) in females compared to males. For the age at first occurrence of IFG, IGT or IFG+IGT, no significant differences between genders were observed.

### 3.3 Risk Factors for Abnormalities in Glucose Metabolism

Table 2 presents Cox proportional-hazards ratios with 95% CIs for either a pathological OGTT or a diagnosis of CFRD in CF patients. Female sex was a risk factor for all abnormalities in glucose metabolism. For the occurrence of DGT and CFRD, the influence was statistically significant. Compared to males, females had a 3.5 fold higher risk for CFRD. Besides female sex, underweight was a significant risk factor for IGT and IFG+IGT. If females and males were analyzed together, underweight had no significant influence on DGT or CFRD.

**Table 2:** Cox proportional-hazards ratios for either a pathological OGTT or a diagnosis of CFRD.

Variables		Hazard ratio [95% confidence interval]	P-value
IFG			
	Sex (female vs. male)	1.12 [0.82–1.54]	NS
	Underweight (yes vs. no)	1.13 [0.94–1.36]	NS
	Age*sex	1.01 [0.99–1.02]	NS
	Underweight*sex	1.05 [0.81–1.36]	NS
IGT			
	Sex (female vs. male)	1.27 [0.84–1.93]	NS
	Underweight (yes vs. no)	1.38 [1.11–1.71]	0.003
	Age*sex	1.01 [0.99–1.03]	NS
	Underweight*sex	0.96 [0.70–1.31]	NS
IFG+IGT			
	Sex (female vs. male)	1.31 [0.80–2.16]	NS
	Underweight (yes vs. no)	1.43 [1.11–1.83]	0.006
	Age*sex	1.01 [0.99–1.03]	NS
	Underweight*sex	0.89 [0.62–1.29]	NS
DGT			
	Sex (female vs. male)	2.21 [1.22–3.98]	0.009
	Underweight (yes vs. no)	1.31 [0.98–1.75]	0.07
	Age*sex	1.03 [1.00–1.05]	0.06
	Underweight*sex	1.14 [0.74–1.75]	NS
CFRD			
	Sex (female vs. male)	3.54 [1.23–10.18]	0.02
	Underweight (yes vs. no)	0.96 [0.58–1.62]	NS
	Age*sex	1.04 [0.99–1.09]	0.10
	Underweight*sex	1.40 [0.66–3.00]	NS

Each model includes sex and underweight as dichotomous variables, and interaction terms for age with sex as well as underweight with sex. Abbreviations: CFRD = cystic fibrosis-related diabetes, DGT = diabetic glucose tolerance, IFG = impaired fasting glucose, IGT = impaired glucose tolerance, OGTT = oral glucose tolerance test, NS = not significant (p>0.10).

In Cox proportional-hazards models stratified by gender, underweight was a stronger risk factor for DGT and CFRD in males compared to females (HR [95% CI] for DGT: 1.49 [1.09–2.04] vs. 1.31 [0.98–1.76]; for CFRD: 1.36 [0.77–2.38] vs. 0.96 [0.57–1.60]). For the occurrence of IFG+IGT, the influence of underweight was stronger in females compared to males (1.44 [1.12–1.85] vs. 1.27 [0.98–1.66]). For less severe abnormalities of glucose tolerance, gender-specific Cox proportional-hazards models elucidated underweight as a risk factor with similar influence for both genders.

## Discussion

The principle objective of our study was to identify CF patients with previously undiagnosed CFRD by OGTT in one of the largest screening cohorts (n = 1,658). This study is the first large one using a stringent definition for CFRD in compliance with current guidelines. A critical literature review revealed that previous studies often used the terms ‘DGT’ and ‘CFRD’ equivalently. However, the current guidelines for CFRD recommend that a diabetic OGTT should be confirmed by a second test, unless patients have unambiguous symptoms of hyperglycaemia [2]. In CF, glucose tolerance varies, depending on individual health status [2]. Hence, in the present analysis, CFRD was diagnosed based on two consecutive diabetic OGTTs.

Only few studies using the recommended definition for CFRD could be identified in the literature. The Canadian CF Patient Data Registry Report describes a rise in CFRD prevalence with age [20]. 25% of patients aged 25 to 34 years had CFRD. Above 35 years, frequency of CFRD amounted to 28.1% [20]. Lanng et al performed OGTTs in 191 patients (median age at study entry 13.6 years) over a 5 year study period [7]. Compared to our patients, age at CFRD diagnosis was lower.

In contrast, most previous studies used the term ‘CFRD’ for patients with one diabetic OGTT (DGT in the present study). In addition, diabetes diagnosis was made using multiple criteria including for example random or fasting glucose levels, or hyperglycemic symptoms besides OGTT. Moran et al investigated 527 pediatric and adult CF patients and found a CFRD prevalence of about 50% in patients aged 30 to 39 years [1]. A Danish study performed OGTTs annually and the authors reported that up to 50% of patients aged ≥30 years have CFRD [8]. A longitudinal follow-up of glucose tolerance in 206 French patients (average age at first OGTT: 12.6 years) [5], showed a lower age at occurrence of diabetes compared to our result (50% of patients: 22.5 years vs. 37.7 years). In part, this can be attributed to different definitions for DGT.

Even with the recommended definition for CFRD based on two consecutive diabetic OGTTs, females had a significantly earlier onset of DGT and CFRD. Furthermore, female sex was a risk factor for all abnormalities in glucose metabolism. Comparisons with the literature are difficult due to various definitions of diabetes. A higher frequency and an earlier onset of diabetes among females were repeatedly reported [1], [3], [5], [21]–[23], while one study found no difference among genders [24]. Besides female sex, underweight was a significant risk factor for the occurrence of IGT and IFG+IGT, and in males also for DGT. A negative effect of undernutrition on glucose metabolism was described previously [22], [25], [26]. Assessment of nutritional status by BMI in our CF patients revealed similar results as studies from Bell et al and Stern et al [27], [28]. A poorer nutritional state in male compared to female adolescents was reported by Bell et al [27].

It is still unclear whether the ADA diagnostic criteria [29] or even lower thresholds [2] should be used in CF patients to define abnormalities in glucose metabolism, especially CFRD. Start and type of insulin therapy depend on blood glucose values (fasting or postprandial) that rise earlier in CF. We showed in our analysis that at the age of 20 years a large proportion of CF patients had FBG elevated ≥5.6 mmol/l. However, if the old WHO criterion of 6.1 mmol/l was used as threshold for elevated FBG, more patients had elevated 2 h-BG levels (≥7.8 mmol/l). Therefore, our results suggest that the question whether FBG or 2 h-BG rise earlier in CF patients depends on the thresholds used. Different thresholds for abnormal glucose metabolism in CF were part of a previous study from our group [13]. A further analysis conducted by our group identified IFG, IGT and one-hour elevated blood glucose (INDET) as predictors for future DGT in CF patients [30].

The strengths of this multicenter screening program are its large size, its long duration and the stringent criteria used for CFRD diagnosis. Possible limitations are the lack of consistent yearly follow-up and the lack of longitudinal data in a part of the cohort. In addition, not all patients with a diabetic OGTT had a second OGTT within the study period. Selection biases are also possible and should be kept in mind. Especially older CF patients might be excluded earlier from screening due to clinical symptoms, accompanying therapies (steroids, lung transplantation, tube feeding), already established CFRD diagnosis or death. Underestimation of diabetes and glucose tolerance abnormalities in older patients is therefore possible, and contributes to higher age at onset of abnormalities in study populations including all ages compared to pediatric or adolescent cohorts. Nevertheless, current data on the age-dependent frequency of glucose abnormalities identified by OGTT screening in a large cohort of German and Austrian CF patients are provided.

## Conclusion

In CF patients screened by OGTT, an early onset of abnormalities in glucose metabolism that became more frequent with age was observed. Using the recommended definition, age at CFRD diagnosis was higher than reported by most previous studies. At 20 years of age, about 10.5% of patients screened by OGTT were identified having CFRD. Risk factors like female sex and underweight were confirmed by this study. In male CF patients, underweight seemed to be a stronger risk factor for DGT and CFRD compared to females. To our best knowledge such a finding has not been reported yet. The general assumption of normal FBG levels for a long period of time in CF depends on threshold values used. With the new ADA recommended threshold of 5.6 mmol/l, elevated FBG levels were more common than elevated 2 h-BG levels. Confirmation of diabetic OGT by a repeat test is important for a consistent diagnosis of CFRD in compliance with current guidelines. Future research should consider the current guideline recommendations for CFRD diagnosis and treatment.

## Acknowledgments

The authors thank Prof. Dr. Elisabeth Kohne (Department of Pediatrics and Adolescent Medicine, University Hospital Ulm) for performing HbA1c measurements. In addition, they wish to express their gratitude to all participating centers for performing OGTTs and sharing data for the present analysis:

Klinik für Kinder- und Jugendmedizin Berlin, Helios Klinikum Berlin-Buch Klinik für Kinderheilkunde/Jugendmedizin, Helios Klinikum Emil von Behring Campus Benjamin Franklin Berlin, Evang. Krankenhaus Bielefeld Kinderklinik in Bethel, Universitätskinderklinik Bochum, Prof.-Hess-Kinderklinik Bremen, Zentralkrankenhaus “Links der Weser” Klinik für Kinder- und Jugendmedizin Bremen, Universitätskinderklinik Düsseldorf, Ruhrlandklinik Essen, Universitätsklinikum GHS Essen, Kinder- und Jugendklinik Universitätsklinikum Erlangen, Med. Klinik II Allergologie und Pneumologie Frankfurt/Main, Zentrum der Kinderheilkunde Frankfurt/Main, Universitätsklinikum Freiburg Zentrum für Kinderheilkunde und Jugendmedizin, Georg-August-Universität Göttingen Kinder- und Poliklinik, Universitätsklinik für Kinder- u. Jugendheilkunde Graz, Klinik und Poliklinik für Kinder- und Jugendmedizin der Universität Greifswald, Martin-Luther-Universität-Wittenberg Klinikum Kröllwitz Kinderklinik Halle, Martin-Luther-Universität-Wittenberg Klinikum Kröllwitz Zentrum für Innere Medizin Halle, Altona - Klinik Hamburg, Medizinische Hochschule Hannover Abt. Kinderheilkunde 1, Medizinische Hochschule Hannover Pneumologische Ambulanz, Klinikum der Universität Heidelberg Kinderklinik Abt. III, Thoraxklinik Heidelberg Innere Medizin, Universitätsklinik für Kinder- u. Jugendmedizin Homburg/Saar, Mukoviszidosezentrum der Friedrich-Schiller-Univ. an der Klinik für Kinder-und Jugendmedizin Jena, Kinderkrankenhaus Park Schönfeld Kassel, Städtisches Krankenhaus/Klinik für Kinder- u. Jugendmedizin Kiel, Klinikum der Universität zu Köln Klinik und Poliklinik für Allg. Kinderheilkunde, Universitätsklinik Mainz, Klinik für Kinder- und Jugendmedizin Clemenshospital Münster, Klinikum Neubrandenburg Kinderklinik, Kinderklinik Offenburg, Elisabeth-Kinderkrankenhaus Oldenburg, Kinderhospital Osnabrück, Kinderklinik Dritter Orden Passau, Landesklinik für Kinder- und Jugendheilkunde Salzburg, Universitätsklinik für Kinder- und Jugendmedizin Tübingen, St. Marienhospital Kinderklinik Vechta, Dt. Klinik für Diagnostik Wiesbaden, Kinderklinik des Reinhard-Nieter-Krankenhauses Wilhelmshaven, Julius-Maximilians-Universität Kinderpoliklinik Würzburg, Universitätsklinik für Kinder- und Jugendheilkunde Wien.
